# Double Pulse Resistance Spot Welding of Dual Phase Steel: Parametric Study on Microstructure, Failure Mode and Low Dynamic Tensile Shear Properties

**DOI:** 10.3390/ma14040802

**Published:** 2021-02-08

**Authors:** Imtiaz Ali Soomro, Srinivasa Rao Pedapati, Mokhtar Awang

**Affiliations:** Department of Mechanical Engineering, Universiti Teknologi PETRONAS, Seri Iskandar 32610, Malaysia; imtiaz_17007503@utp.edu.my (I.A.S.); srinivasa.pedapati@utp.edu.my (S.R.P.)

**Keywords:** dual phase steel, resistance spot welding, in situ postweld heat treatment, tensile shear test, fusion zone, martensite, Taguchi design

## Abstract

Resistance spot welding (RSW) of dual phase (DP) steels is a challenging task due to formation of brittle martensitic structure in the fusion zone (FZ), resulting in a low energy capacity of the joint during high-rate loading. In the present study, in situ postweld heat treatment (PWHT) was carried out by employing a double pulse welding scheme with the aim of improving the mechanical performance of DP590 steel resistance spot weld joint. Taguchi method was used to optimize in situ PWHT parameters to obtain maximum peak load and failure energy. Experiments were designed based on orthogonal array (OA) L16. Mechanical performance was evaluated in terms of peak load and failure energy after performing low dynamic tensile shear (TS) test. Microstructural characterization was carried out using a scanning electron microscope (SEM). The results show that improvements of 17 and 86% in peak load and failure energy, respectively, were achieved in double-pulse welding (DPW) at optimum conditions compared to traditional single-pulse welding (SPW). The improvement in mechanical performance resulted from (i) enlargement of the FZ and (ii) improved weld toughness due to tempering of martensite in the FZ and subcritical heat affected zone (SCHAZ). These factors are influenced by heat input, which in turn depends upon in situ PWHT parameters.

## 1. Introduction

Dual phase (DP) steels are members of the advanced high-strength steel (AHSS) group and are widely used in vehicle body structure due to their great potential to simultaneously improve fuel efficiency and crash resistance, while offering thin gauge sheets to reduce vehicle weight [1]. DP steels possess high strength and excellent ductility. Apart from that, high strain hardening rate, continuous yielding behavior and low yield to tensile strength ratio are other significant features of DP steels. The combination of these superior mechanical properties is the synergistic effect of their unique microstructure, composed of a soft ferrite matrix embedded with hard martensite islands [2,3].

Vehicle body components are made of thin sheet parts that are joined using resistance spot welding. Several thousand spot welds are made in a modern vehicle [4]. In the event of a crash, passenger safety against injuries largely depends on the vehicle structural integrity, which in turn depends upon the performance of spot welds. During a crash, a spot weld acts as a fold initiation site and transfers the load to automotive assemblies. Therefore, for the safe design of the vehicles, the performance and quality of resistance spot welds must be taken into account [5,6].

One of the key quality characteristics affecting the mechanical performance of spot welds is the mode of failure. Generally, a spot weld fails in two modes, i.e., pullout failure mode (PF) and interfacial failure (IF) mode. PF mode, in which the failure occurs via withdrawal of the weld nugget from sheets, exhibits the most satisfactory mechanical properties. On the contrary, IF mode (in which a fracture propagates through the FZ) is detrimental for vehicle crashworthiness and must be avoided. During a crash event, PF mode can transmit a high level of force and increase the strain energy dissipation, thus causing severe plastic deformation in its adjacent components [7,8].

Generally, the failure behavior of a spot weld joint is determined based on a static collapse process using static loading conditions through laboratory tests, i.e., coach-peel test, lap-shear tensile test and cross-tension test. However, in real a crash situation, the behavior of a joint can be very different to that of the statically loaded laboratory tested sample. During a crash event, a large load is suddenly transferred to the vehicle structure and extremely high stress is concentrated at the weld nugget/base metal (BM) interface. Consequently, spot weld failure is likely to occur prior to failure of the BM [9]. In order to perform a critical analysis of the crashworthiness of vehicle structural members, it is imperative to understand the spot weld failure behavior under dynamic loading rates. For this purpose, rapid loading tests such as the drop weight test, accelerated tensile shear and cross tension test (CT) and modified impact pendulum test are used [10,11,12]. Chao et al. [13] studied the strain rate sensitivity of resistance spot-welded joints in a tensile shear and cross tension specimen under tensile loading. Dynamic and static test data from a wide range of steel grades, including conventional mild steel, high-strength low-alloy (HSLA) steel and DP steel with different sheet thicknesses and weld button sizes, were recorded. They showed that (a) TS specimen exhibits higher strain rate sensitivity and is stiffer than CT specimen, (b) higher impact speed results in higher loading rate, (c) higher impact speed gives higher fracture load and (d) a higher loading rate can be achieved in an TS specimen. Birch and Alves [14] also conducted quasi-static and dynamic tensile shear testing of spot-welded sheets. The results showed that fracture load increased with increasing test velocity, while failure energy showed dependence on failure mode in addition to test velocity. Song et al. [9] investigated the dynamic failure behavior of the spot weld under combined axial and shear loading using a designed fixture to hold the LS specimen. Various test velocities (i.e., 1 × 10^−5^, 0.01, 0.1 and 1.2 m/s) and loading angles (i.e., 0, 15, 30, 45, 60, 75 and 90°) were applied. It was found that peak load reduced when the loading angle was less than 30°, whereas peak load increased at the interval from 45 to 90° conditions. Moreover, a maximum of 13% improvement in peak load was reported with increasing test velocity from lowest (quasi-static) to highest (dynamic) loading rates.

It is well known that the thermal effect of resistance spot welding destroys the carefully designed multiphase microstructure of advanced high-strength steels (AHSSs), including DP steel. As a result of high hardenability due to increased alloying content and high cooling rates (in the order of 2000–4000 K s^−1^) of the weld during RSW, a hard martensitic microstructure is formed in the FZ and coarse-grain heat-affected zone (CGHAZ) [15,16,17]. Moreover, softening occurs in the SCHAZ due to the tempering of martensite already present in the base metal [18]. Due to the above phase transformations, a significant property (strength and toughness) mismatch occurs among the FZ, HAZ and BM. Consequently, the mechanical properties are impaired locally, resulting in poor joint performance. Khan et al. [11] studied the effect of weld microstructure on the peak load and failure energy absorption of spot welds of conventional HSLA steel, 590R, DP600, DP980 and TRIP780 steel using static, intermediate and dynamic test velocities. They observed that DP600 steel spot weld failed in IF mode for all three test velocities. They reported that poor fracture toughness of the FZ and HAZ due to formation of a martensitic structure, along with the stress concentration at the interface, resulted in low failure loads at all test velocities.

It has been reported that microstructure within the spot weldments can be altered by in situ postweld heat treatment using double pulse welding scheme, and improved mechanical properties can be obtained [15,16,17,19,20,21,22]. In this method, a weld nugget is formed after the primary/first pulse current due to heat generated at the sheet/sheet interface, which in turn melts the sheets. Some cooling time is then allowed to solidify the weld nugget. After that, the weldment is reheated by applying a second pulse current for a certain time duration, with aim to reduce FZ hardness at a sufficient extent, and then cooled again. Apart from microstructure modification, a better weldability range and enhanced weld nugget size can be obtained using resistance spot welding with a multi-pulse current pattern [23,24]. It has been reported that the correct amount of heat input is key to obtaining the desired microstructure in the weldment, which can be controlled by using appropriate in situ PWHT parameters, i.e., second pulse welding current, second pulse welding time and cooling time (time between first pulse and second pulse) [16]. Heat input depends upon welding current and welding time, and heat dissipation (i.e., cooling rate) is influenced by cooling time during RSW. In previous research studies detailing the double pulse RSW, the effect of a second pulse current and time on microstructure and mechanical properties under static loading rates has been studied while effect of cooling time is not investigated well. However, it has been reported that cooling time has a significant effect on the weld microstructure and mechanical properties of AHSS resistance spot welds [25]. Therefore, optimization of all three in situ PWHT pulse parameters including welding current, welding time and cooling time is required to obtain the best combination of mechanical properties.

In this paper, the effect of in situ PWHT parameters, i.e., second pulse welding current, second pulse welding time and cooling time, on microstructure, mechanical properties, and failure modes of DP590 steel RSW is investigated. Mechanical properties in terms of peak load and failure energy were obtained using low a dynamic tensile shear (TS) test (loading rate 500 mm/min). The Taguchi design of experiment (DOE) method is used to determine the optimum parameter levels for achieving the highest TS peak load and failure energy. In addition, significant process parameters affecting the mechanical properties of welds were analyzed by using the analysis of variance (ANOVA) method.

## 2. Materials and Methods

### 2.1. Material

The material used in this study is a galvanized dual phase (DP590) steel sheet with 1.8 mm thickness manufactured by Baoshan Iron & Steel Co., Ltd. PR Baoshan District, Shanghai, China. Mechanical properties along with chemical composition and carbon equivalent (CE) of the investigated steel are given in Table 1.

### 2.2. Resistance Spot-Welding Procedure

**Figure 1:**
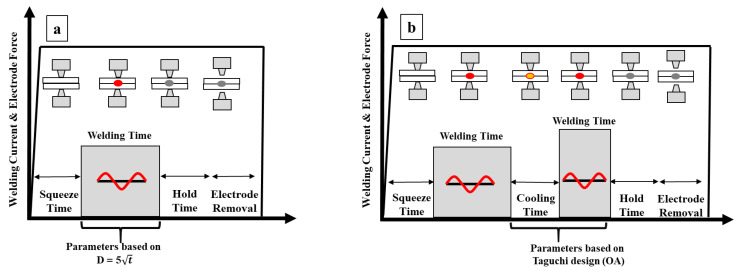
Resistance spot welding (RSW) schemes (**a**) SPW; (**b**) double-pulse welding (DPW). (Reprinted from [26] with permission from Elsevier).

A semi-automatic, alternating current (AC)-type RSW machine (*WIM* JPC 100, manufactured by Welding Industries Malaysia Sdn.Bhd, Pusing, Perak, Malaysia) was used to make spot welds on the specimen. Truncated cone shaped electrodes made of Cu-Cr alloy (Group A, RWMA class 2.18200 and type no.5) with 8 mm face diameter were used for welding. Two welding schemes (refer Figure 1) were used for the present study, namely (i) single pulse welding (SPW) and (ii) double pulse welding (DPW). For SPW, parameters that give a minimum size of spot weld FZ based on the D = 5√t (where D is the width of FZ and t is sheet thickness) criterion recommended for AHSS steel were selected [4]. Table 2 shows the parameters of SPW.

#### Design of Experiments (DOE) for DPW

The three main input parameters, i.e., welding current, welding time and cooling time with four levels were considered for the DPW scheme are shown in Table 3. Taguchi’s experimental design consisting of OA L16 shown in Table 4 was constructed by using Minitab^®^ (18.0) software. The details of the selection of parameter levels are discussed in ref [26].

### 2.3. Metallography

Spot-weld specimens were first sectioned from the weld centerline for metallographic examination. Specimens were then rough polished and fine polished according to standard metallographic procedure, followed by etching in 2 pct Nital reagent. Next, macrostructure and microstructure were examined using a stereomicroscope and scanning electron microscope (SEM, TESCAN VEGA3, Kohoutovice, Czech Republic), respectively.

### 2.4. Mechanical Testing and Microhardness

**Figure 2:**
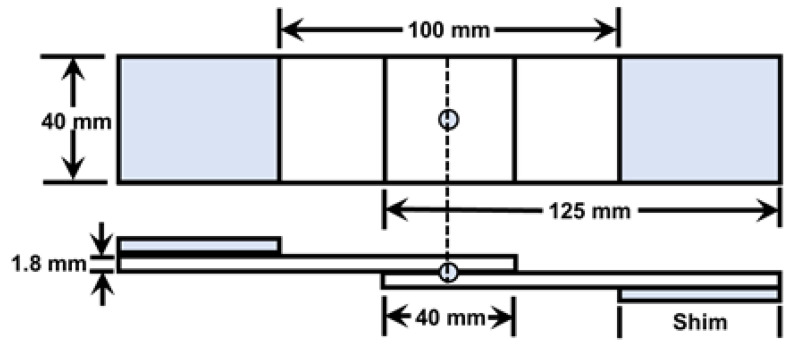
Schematic of lap shear tensile specimen.

Room temperature low dynamic TS testing was performed at a crosshead speed of 500 mm/min using a servohydraulic testing machine (model: ZwickRoell HA50, Zwick GmbH & Co. KG, Ulm, Germany). TS testing was performed on a lap shear tensile specimen prepared according to the JIS Z-3136 (1999) standard as shown in Figure 2. 1.8 mm thick shims were added at the grip sections of the specimen to reduce the sheet bending and nugget rotation. Mechanical properties, i.e., peak load and failure energy, were determined from the load–displacement curve using OriginPro^®^ 2018 software. Failure modes of the weld joints were observed after TS testing using digital images. Vickers microhardness testing was performed with an applied load of 200 g for a loading time of 15 s to measure the hardness across the weld joint.

## 3. Results and Discussion

### 3.1. Microstructure and Microhardness of SPW

**Figure 3:**
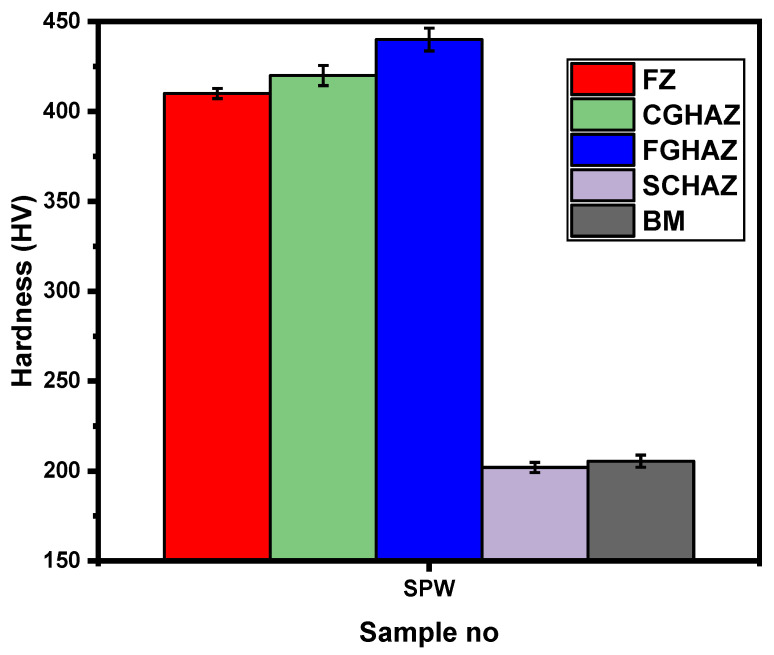
Average microhardness of different weld zones of SPW.

**Figure 4:**
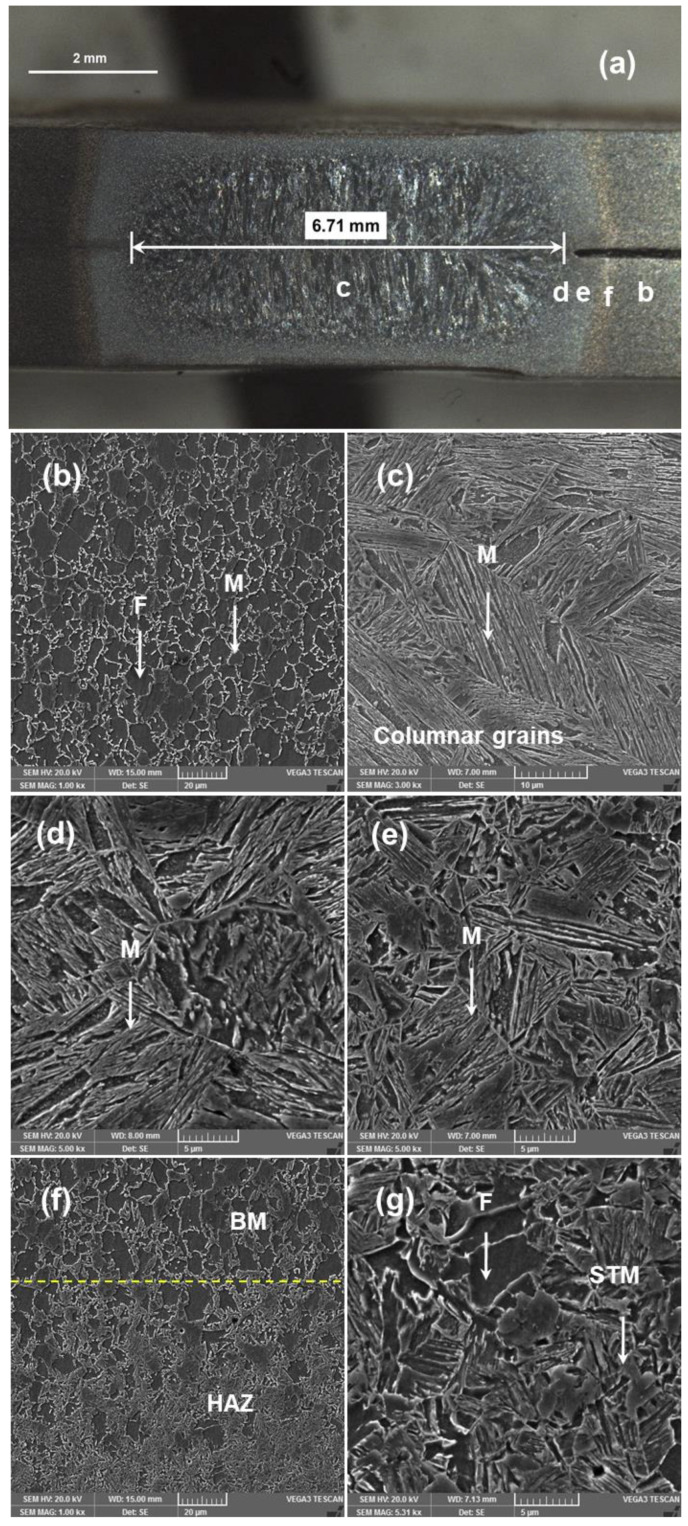
Microstructure evolution of SPW. (**a**) overall weld structure, (**b**) base metal (BM), (**c**) fusion zone (FZ), (**d**) coarse-grain heat-affected zone (CGHAZ), (**e**) fine-grain heat-affected zone (FGHAZ), (**f**) interface between BM and HAZ (**g**) SCHAZ (note F is ferrite, M is martensite, STM is slight tempered martensite).

Figure 3 shows the average microhardness and Figure 4 shows the microstructure evaluation of SPW. Generally, resistance spot weldment has a heterogeneous microstructure in line with the weld thermal cycle. Therefore, weldment can be divided into three regions, namely BM, FZ and HAZ. It is well known that the microstructure of the RSW has a significant effect on microhardness. Therefore, hardness variation across the weldment is explained based on microstructure development. Figure 4a shows the overall macrostructure of SPW indicating that FZ size is 6.71 mm, meeting the minimum weld size requirement based on D = √5t. Figure 4b shows the microstructure of the BM consisting of two phases, i.e., ferrite and martensite, with average hardness ~205 HV. Figure 4c shows the microstructure of the FZ consisting of large columnar grains due to directional solidification of liquid metal towards the weld centerline. The columnar grains mainly consist of lath martensite (hardness ~410 HV). Martensite formation in the FZ can be attributed to the high cooling rate of the RSW process due to the presence of water-cooled copper electrodes and the high hardenability of BM (i.e., C.E = 1.19) [4]. Depending upon grain size, temperature and microhardness distribution, the microstructure of the HAZ is further subdivided into coarse-grain heat-affected zone (CGHAZ), fine-grain heat-affected zone (FGHAZ) and subcritical heat-affected zone (SCHAZ). The microstructure of the CGHAZ is composed of coarse grains of martensite as shown in Figure 4d. During welding, the peak temperature in CGHAZ reaches well above Ac3 (100% austenite region). Upon cooling, both high cooling rate and carbon-rich austenite promote the formation of the coarse grain martensite within large prior austenite grains (PAGs) (hardness ~420 HV). Figure 4e shows that the microstructure of the FGHAZ consists of fine packets of martensite (hardness ~440 HV). During welding, the peak temperatures in the FGHAZ reach slightly above Ac3, which promotes austenite nucleation, while the short heating time and rapid cooling limits grain growth. Figure 4f shows the interface produced between the BM and the HAZ. Figure 4g shows that the microstructure of the SCHAZ is composed of ferrite and slightly tempered martensite (hardness ~202 HV). The peak temperature becomes less than the Ac1 temperature with increasing distance from the fusion boundary, resulting in tempering of the martensite phase present in BM [6]. However, no significant softening was observed in the SCHAZ. This can be attributed to the lower martensite content of BM and the lower heat input of the first pulse current.

**Figure 5:**
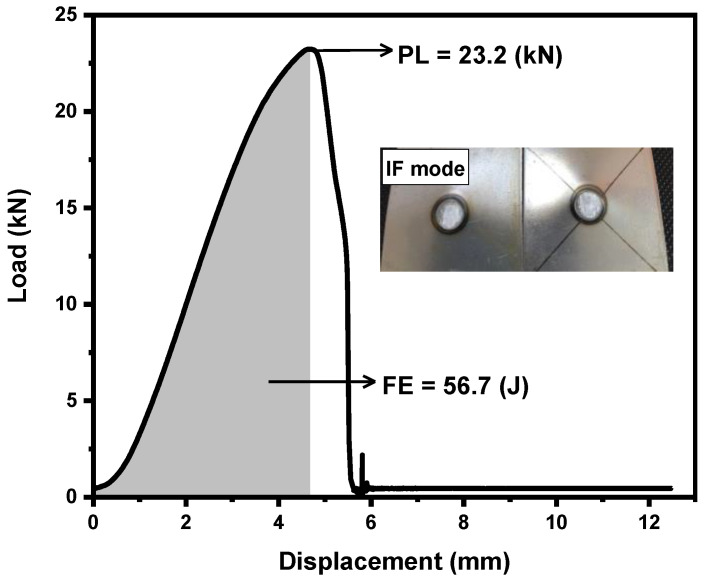
Load–displacement plot of SPW indicating peak load (PL) and failure energy (FE).

Figure 5 shows the load–displacement plot obtained after TS testing. It can been seen that peak load and failure energy were 23.2 kN and 56.7 J, respectively. Figure 5 also illustrates the failure mode of SPW after TS testing, indicating the IF mode (also confirmed by a sudden drop of load in the load–displacement curve). The IF mode in SPW can be attributed to the formation of a brittle martensitic microstructure in the FZ and HAZ. The IF mode of SPW implies that a minimum sizing criterion based on D = 5√t rule does not guarantee PF. Therefore, it seems that in addition to sheet thickness, the microstructural characteristics of the weldment influence the failure mode.

### 3.2. S/N Ratio Analysis for DPW

In general, the Taguchi method adopts the signal-to-noise (S/N) ratio to evaluate the performance characteristics. The S/N ratio is a logarithmic function of the desired performance characteristics that serves as an objective function for the optimization [28]. For evaluating performance characteristics three approaches, i.e., larger-the-better, smaller-the-better and nominal-the-better, are used. For obtaining maximum peak load and failure energy, the larger-the-better approach is used to calculate S/N ratio using Equation (1).

(1) $$\left(\frac{S}{N}\right)ratio=-10\log\left(\frac{1}{n}\sum_{i=1}^{n}\frac{1}{y_{i}^{2}}\right)$$

where $y_{i}$ represents the response value of the ith experiment in the orthogonal array and n is the number of experiments. Each test was repeated one time and the mean values of the response characteristics (i.e., peak load and failure energy) were computed. The experimental results of the response characteristics along with corresponding S/N ratios are given in Table 5.

In the Taguchi method, response curves are used for examining the effect of input parameters on the response characteristics. In the present study, the most favorable conditions (optimal setting) for process parameters in terms of mean response characteristics are established by analyzing response curves. Moreover, analysis of variance (ANOVA) was performed at the 95% confidence level to estimate quantitatively the relative significance and the percentage contribution of input control factors on response characteristics [28].

#### 3.2.1. Optimum In Situ PWHT Parameters for Maximum Peak Load

**Figure 6:**
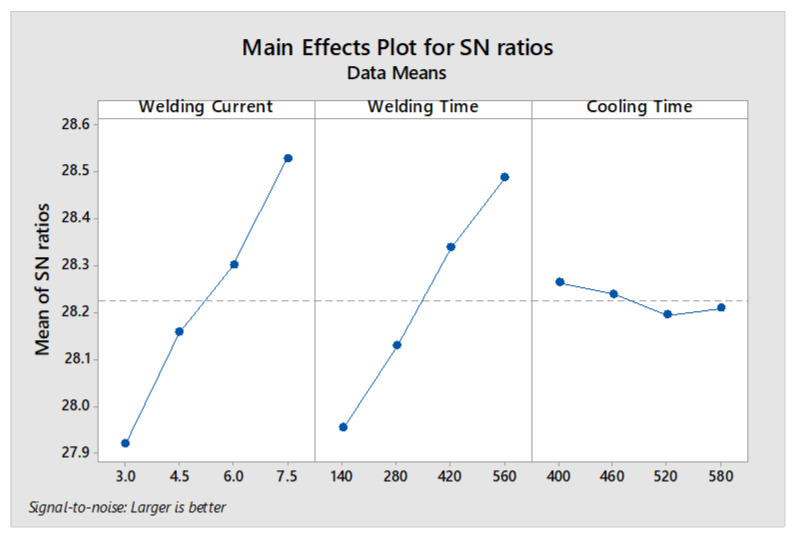
Graph representing main effects for peak load.

Figure 6 shows that the optimum parameters for maximum peak load are A4B4C1, i.e., WC (7.5 kA), WT (560 ms) and CT (400 ms). It is noteworthy here that the optimum conditions for peak load coincidently match with sample 16. Table 6 shows level rankings based on S/N ratios of the three parameters. The higher the delta value of a parameter, the higher the rank and the more influential that parameter would be. According to Table 6, the highest delta value is 0.61, which indicates that welding current has a greater effect on peak load compared to welding time and cooling time. ANOVA results are given in Table 7. According to ANOVA, welding current and welding time have a significant effect on peak load. Moreover, based on the percentage of contribution, the most dominating parameter is welding current, followed by welding time and cooling time.

#### 3.2.2. Optimum In Situ PWHT Parameters for Maximum Failure Energy

**Figure 7:**
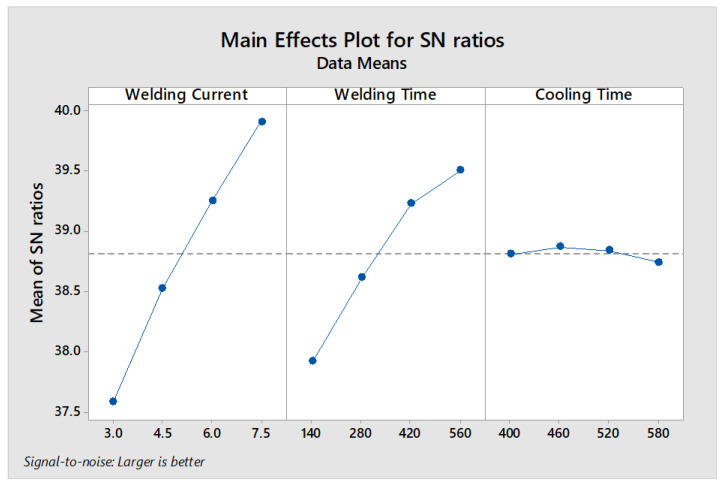
Graph representing main effects for failure energy.

For maximum failure energy, the optimum parameter levels are A4B4C2, i.e., WC (7.5 kA), WT (560 ms) and CT (460 ms) as shown in Figure 7. Table 8 shows the ranking of parameters for S/N ratio of failure energy. According to Table 6 the highest delta value is 2.33, which indicates that welding current has a greater effect on failure energy compared to welding time and cooling time. ANOVA results are given in Table 9. According to ANOVA, welding current and welding time are significant factors. Moreover, based on the percentage of contributions, the most dominating parameter is welding current followed by welding time and cooling time. Based on S/N ratio analysis, the predicted value of failure energy at A4B4C4 can be calculated using the equation given below.

(2) $$\eta_{opt}=\hat{\eta }+\sum_{j=1}^{q}\left(\eta_{j}-\hat{\eta }\right)$$

where $\eta_{j}$ is the mean S/N ratio at the optimal level, $\hat{\eta }$ is the average of the S/N ratios of all the experimental values of failure energy and q is the number of parameters that significantly affect the failure energy (two parameters, i.e., WC and WT). According to Equation (2), the predicted value of failure energy at A4B4C2 is ~106.07 (J).

### 3.3. Effect of In Situ PWHT Parameters on Mechanical Properties of DPWs

Generally, the mechanical properties of the spot weld are described in terms of peak load and failure energy. Three quality measures influence the peak load and failure energy, including the strength and ductility of failure location, which in turn depend upon the microstructure of the FZ/HAZ, geometrical weld attributes (mainly FZ size) and the failure mode (depends upon both FZ size and strength of failure location) [29]. According to experimental results (refer to Table 5), DPW shows higher values of peak load and failure energy compared with SPW. Based on average values, it was found that maximum improvement of 17% in peak load and 86% in failure energy are achieved in sample 16 compared with SPW. Therefore, the improvement in mechanical properties is explained based on the above three quality measures.

#### 3.3.1. Microstructure and Microhardness

**Figure 8:**
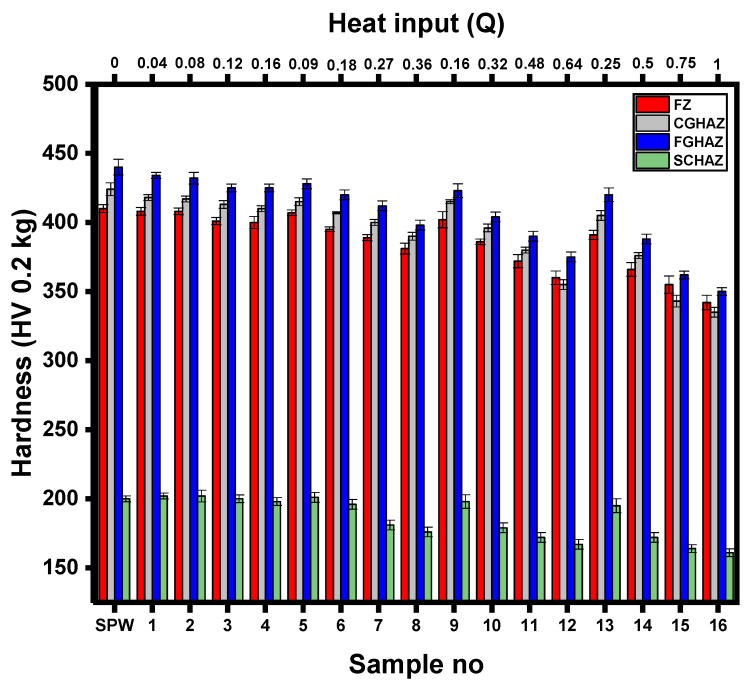
Average hardness profile of SPW and DPWs.

**Figure 9:**
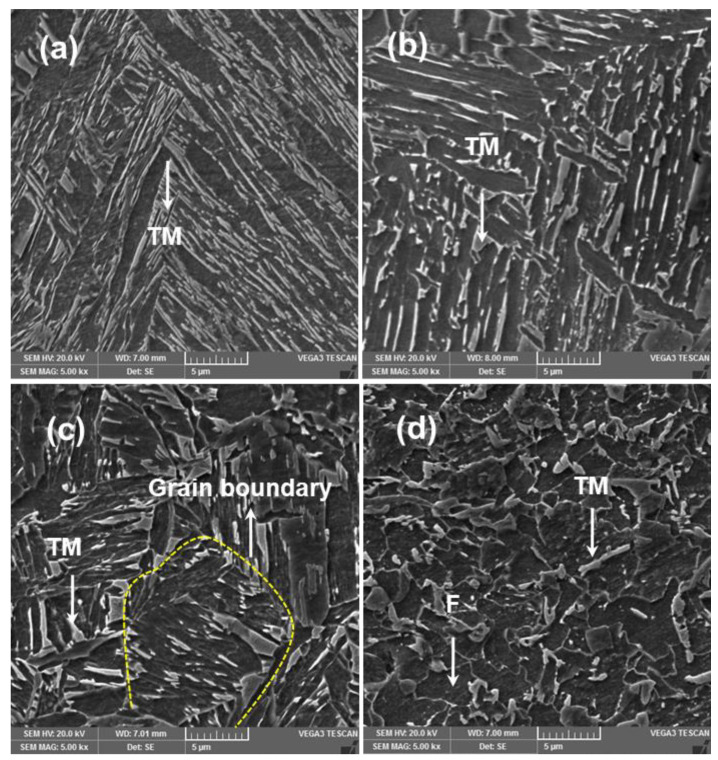
Microstructure evaluation of DPW16 RSW. (**a**) FZ, (**b**) CGHAZ, (**c**) FGHAZ and (**d**) SCHAZ.

Figure 8 illustrates the effect of heat input on the average microhardness distribution of all welds. It can be observed that microhardness increases with SCHAZ, FZ, CGHAZ and FGHAZ. It is noteworthy here that SPW exhibits the highest microhardness in all weld zones compared to all DPW samples. In DPWs, the microhardness of the FZ and HAZ substructures depends upon microstructural phases, which are in turn influenced by heat input during the RSW thermal cycle. Heat input for DPW was calculated using Equation (3) discussed in Section 3.3.2. According to Figure 8, sample 16 experienced the maximum heat input during in situ PWHT. Moreover, according to DOE statistics, sample 16 showed the highest values of peak load and failure energy. Therefore, for simplicity, variation of hardness in sample 16 is explained in terms of microstructure development. Figure 9 shows the microstructure evolution of different weld zones of sample 16. It can be observed that the microstructure of FZ (refer to Figure 9a) exhibits broken laths of martensite within large columnar grains, resulting in reduced hardness (16.5% lower than the FZ of SPW). Moreover, the microstructures of HAZ substructures, i.e., CGHAZ (refer to Figure 9b), FGHAZ (refer to Figure 9c) and SCHAZ (refer to Figure 9d), also show severely tempered martensitic structures, indicating a broken lath morphology within prior martensite grains resulting in hardness reductions of 20.4, 20.2 and 20.2%, respectively, compared with the CGHAZ, FGHAZ and SCHAZ of SPW. The broken lath morphology in different zones of the sample 16 spot weld can be attributed to partial recovery of martensite laths due to high heat input during the rapid tempering process. Partial recovery of the martensite laths during rapid tempering of DP steel was also reported by Hernandez et al. [18,30]. Partial recovery during rapid tempering is a combined effect of (i) retarding of the lath boundary by fine and dense cementite precipitation on dislocations and (ii) insufficient time for the annihilation of dislocations by complete recovery [27,30,31]. As mentioned earlier, the microstructure and hardness characteristics of different zones of RSW have a significant effect on failure behavior. Therefore, a detailed discussion on the effect of microhardness on failure mechanisms is given in Section 3.3.3.

#### 3.3.2. Enlargement of FZ

**Figure 10:**
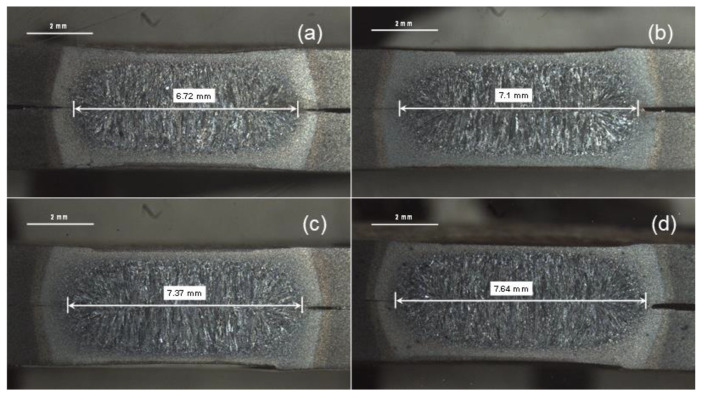
Macrostructure illustrating FZ size. (**a**) sample 1, (**b**) sample 8, (**c**) sample 12 and (**d**) sample 16.

**Figure 11:**
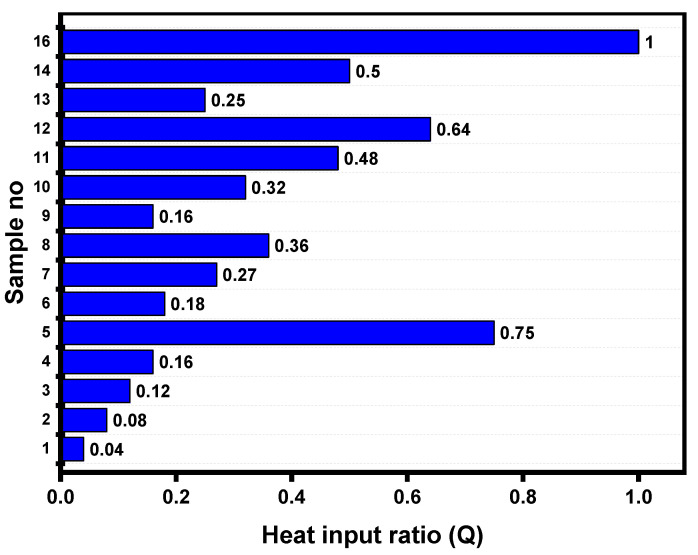
Heat input ratio of DPWs. (Reprinted from [26] with permission from Elsevier)

**Figure 12:**
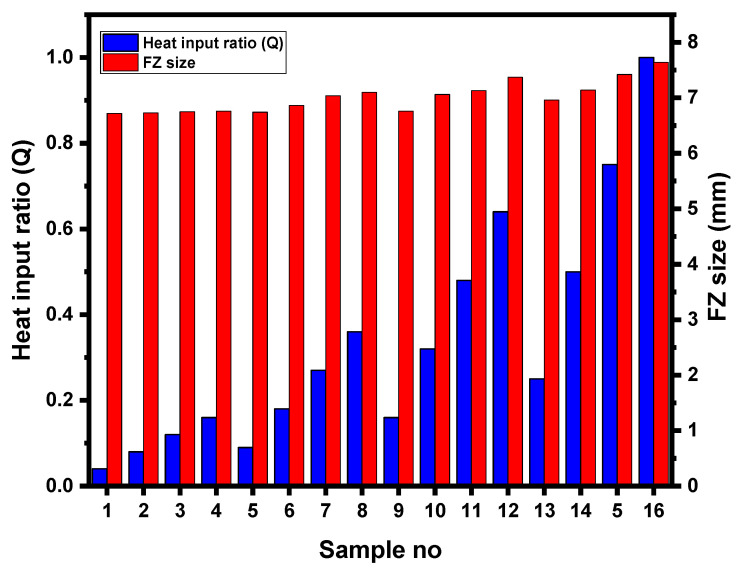
Effect of heat input on FZ size.

FZ size is one of the most important qualitative characteristics affecting the mechanical performance of spot weld joint [8]. The macrostructure indicating the FZ size of samples 1, 8, 12 and 16 is shown in Figure 10. It can be observed that FZ size of samples subjected to DPW scheme is higher compared to SPW. As the FZ size increases, the bond area between the two sheets increases, which in turn improves the peak load and failure energy of welds [8]. According to the main effects plot (refer to Figure 6 and Figure 7), as the second pulse welding current and welding time increase, peak load and failure energy increase. The FZ size depends upon the amount of heat generated during RSW. The physical principal for achieving the heat generation in the RSW process is defined by Joule’s heating equation given below [32].

(3) $$Q=\int_{0}^{t}I^{2}Rt$$

where Q is the heat generated (J), I is the current applied (A), R is the total resistance (Ω) and t is the time for which current is applied (s). Equation (3) indicates that the amount of heat generated increases as the current and time increase. Moreover, current makes a higher contribution to heat generation due to its square value in Equation (3). The weld nugget is formed after applying the first pulse current following the SPW scheme, leading to an FZ size of 6.71 mm. In the DPW scheme, the weldment was reheated using various second pulse current and time combinations after allowing a specific cooling time between the two pulses. The experimental determination of heat input in weldment during the in situ PWHT schedule is difficult due to invisible weld joints and the short welding cycle of RSW. Taniguchi et al. [24] calculated the heat input (Q) ratio of a postweld heating pulse during RSW using the equation given below.

(4) $$Q={\left(\frac{I_{S}}{I_{F}}\right)}^{2}\left(\frac{t_{S}}{t_{F}}\right)$$

where Q is heat input ratio, $I_{S}$ is second pulse welding current (kA), $I_{F}$ is first pulse welding current (kA), $t_{S}$ is second pulse welding time (ms) and $t_{F}$ is first pulse welding time (ms). Figure 11 shows the heat input ratio for all DPW schedules as defined in Table 4. It can be observed from Figure 11 that the value of Q increases with increasing $I_{S}$ and $t_{S}$. Therefore, the variation in FZ size can be explained based on heat input during the second pulse current. Figure 12 shows the effect of heat input on FZ size. It can be observed that FZ size increases as the heat input increases. Moreover, Figure 6 and Figure 7 show that the optimum cooling times for maximum peak load and failure energy are 400 and 460 ms, respectively (i.e., lower levels of cooling time). This indicates that a certain amount of heat is retained in the weldment at the end of cooling, which supplements the total heat input. Aghajani et al. [21] and Lee et al. [33] reported similar observations that the FZ size increases using double pulse RSW.

#### 3.3.3. Failure Mode

**Figure 13:**
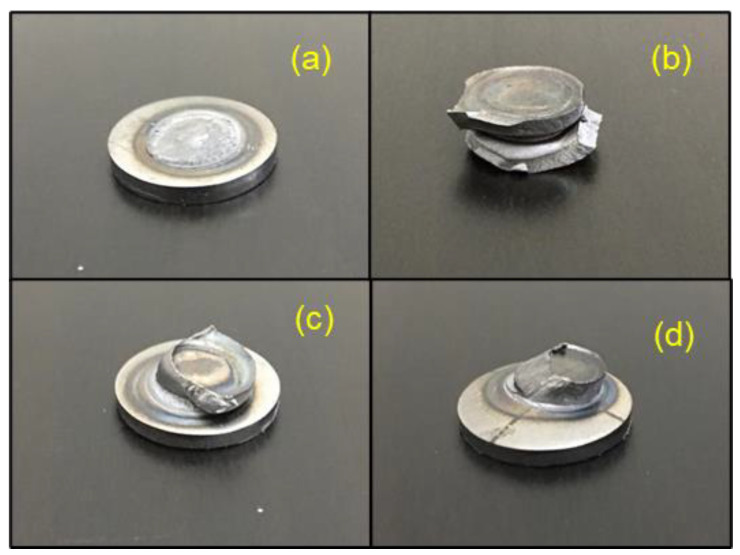
Typical failure modes observed after low dynamic TS testing. (**a**) Interfacial failure (IF) mode, (**b**) pullout failure (PF) mode, (**c**) partial interfacial failure (PIF) mode and (**d**) partial thickness–partial pullout (PTPP) mode.

It has been reported that spot welds that fail in the PF mode exhibit higher load bearing and failure energy absorption capacity compare to welds that fail in the IF mode [6,7]. After conducting low dynamic TS testing, four distinct failure modes were observed for DPWs as shown in Figure 13. The failure mechanism in each case of failure mode is discussed as follows.

1. Interfacial failure (IF) mode. In this mode a fracture propagates through the FZ and the load suddenly drops to zero due to the rapid progression of the crack through the weld centerline.
2. Pullout failure (PF) mode. In this mode failure occurs via withdrawal of the weld nugget from both sheets. During loading, when there is a certain amount of rotation, the tensile stresses formed around the nugget cause plastic deformation in the sheet thickness direction. Finally, necking occurs at the HAZ as tensile force increases, resulting in complete tearing and shearing of the BM.
3. Partial interfacial failure (PIF) mode. In this mode the fracture first propagates in the FZ and is then redirected through the thickness direction.
4. Partial thickness–partial pullout (PTPP mode). In this mode a slant crack first propagates into the FZ and then the crack is redirected through the thick sheet in the thickness direction, resulting in removal of some part of the mating sheet.

**Figure 14:**
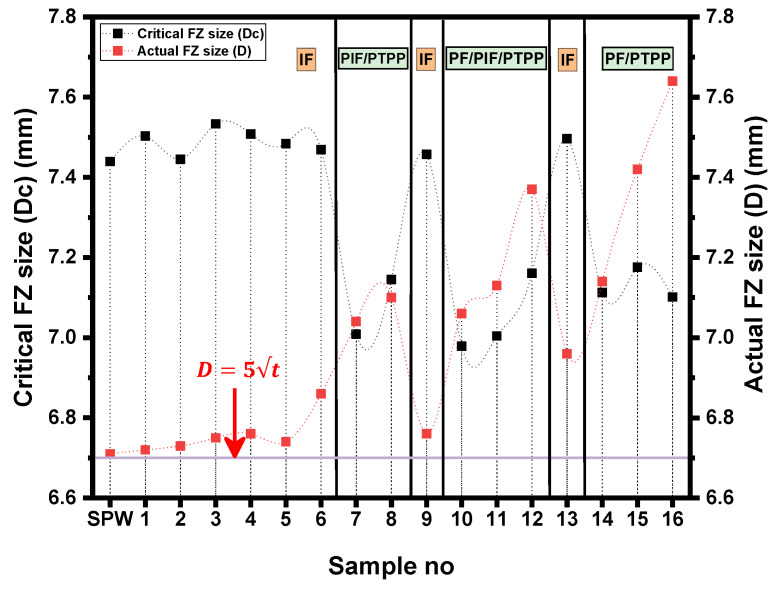
Relationship between critical FZ, actual FZ size and failure mode of SPW and all DPWs.

It has been reported that there is a critical FZ size above which the PF mode occurs during TS loading [34,35]. The critical FZ size depends upon sheet thickness and the mechanical properties of the BM/HAZ/FZ. Under TS loading, for the IF mode the strength of the FZ (which depends upon hardness) is important for predicting the mechanical strength of the weld, and for the PF mode the strength of the failure location (i.e., SCHAZ or BM) determines the mechanical properties of the spot weld [35]. Therefore, under TS loading, the failure of the spot weld is a competition between the shear plastic deformation of the FZ and the necking in the failure location. Necking occurs in the softest region of the weld. Figure 8 shows that the lowest hardness occurs in SCHAZ. Moreover, shrinkage voids/porosity in the FZ affect the failure mode [34,35]. To ensure the PF mode under TS loading, a simple analytical model was developed by Pouranvari et al. [34] and is given below.

(5) $$D_{C}=\frac{4t}{Pf}\frac{H_{PFL}}{H_{FZ}}$$

where Dc is critical FZ size, P is the porosity factor calculated using Equation (6), f is the ratio of shear strength to tensile strength of the FZ and according to the Tresca criterion is equal to 0.5, t is sheet thickness and $H_{FZ}$ and $H_{PFL}$ are the hardness of fusion zone and the hardness of pullout failure location (i.e., SCHAZ), respectively.

(6) $$P=\frac{A_{total}-A_{porosity}}{A_{total}}$$

where $A_{total}$ is the total area of the FZ and $A_{porosity}$ is the projected area of the porosity in the FZ (*P* = 1 if no porosity is present). According to this model, spot welds with Dc<D (where D is actual FZ size and Dc is calculated using Equation (5)) tend to fail via IF mode and spot welds with Dc≥D tend to fail via the PF mode. Figure 14 shows the relationship between critical FZ size, actual FZ size and failure mode. It can be observed that welds (i.e., SPW, sample 1, sample 2, sample 3, sample 4, sample 5, sample 6, sample 9 and sample 13) failed in the IF mode, while other welds failed in the PF/PIF/PTPP modes. Lee et al. [33] also reported that double-pulse RSW results in higher heat input, which in turn affects the weld size, microstructure of the HAZ and failure mode. Increasing heat input during the second pulse intensifies the martensite tempering both in the FZ and the HAZ. However, more softening is observed in the SCHAZ compared to other regions of the weld, which in turn improve the toughness of weldment. It is well known that softening reduces the strength of the SCHAZ and results in strain localization, and hence encouraged the PF mode [6,33]. Moreover, Figure 14 also indicates that the conventional weld size criterion based on D = 5√t is not sufficient to produce the PF mode. Heat input is the dominant factor that causes the failure mode to change from IF to PF during in situ PWHT [33]. Spot welds that experienced lower heat input failed in the IF mode with low peak load and failure energy compared to welds experiencing higher heat input and failure in PF/PIF/PTPP mode.

## 4. Conclusions

Resistance spot weld made on DP590 steel using the SPW scheme showed low load bearing and failure energy absorption capacity. SPW suffers from the IF mode due to formation of brittle martensitic microstructure in the FZ and HAZ. It was observed that applying in situ PWHT via second pulse current in the welding schedule can remarkably enhance the peak load and failure energy of the spot welds at proper second pulse conditions. Based on the experimental results, the following conclusions are drawn:

- At optimum insitu PWHT parameter conditions, the TS peak load and failure energy were enhanced over 17 and 86%, respectively, compared with SPW. The optimum parameter conditions for achieving maximum peak load are A4B4C1, i.e., WC (7.5 kA), WT (560 ms) and CT (400 ms). While the optimum parameter conditions for achieving maximum failure energy are A4B4C2, i.e., WC (7.5 kA), WT (560 ms) and CT (460 ms).
- It was found that welding current is the most dominant factor affecting the mechanical performance of DPWs, followed by welding time and cooling time.
- The improvement in mechanical performance of DPWs is attributed to two factors, i.e., (i) increment of FZ size and (ii) reduction of FZ and HAZ hardness due to tempering of martensite. These factors are influenced by the heat input of the welding process. It was found that after formation of the initial weld nugget, increasing the second pulse current and time increases the heat input, which results in enhanced FZ size. In addition, as the heat input increases, partial recovery of martensite laths was observed in FZ and SCHAZ.
- IF to PF mode transition is correlated to (i) increasing FZ size and (ii) improved toughness of the weldment via encouraging martensite tempering both in the FZ and the SCHAZ.

## Figures and Tables

**Table 1:** Mechanical properties, chemical composition and carbon equivalent (CE) of investigated steel. (Reprinted from [26] with permission from Elsevier)

Chemical Composition									
Al	Si	Cr	P	S	Mn	Mo	Ni	C	Fe
0.0301	0.388	0.003	0.022	0.007	0.70	0.0145	0.0144	0.099	Balance
**Mechanical Properties**							* CE		
**YS (MPa)**			**UTS (MPa)**		**T.E (%)**		1.19		
440			694		11.58				

* CE of steel was calculated using the Yurioka [27] formula.

**Table 2:** Parameters and their levels for the single pulse welding (SPW) scheme. (Reprinted from [26] with permission from Elsevier)

ST (ms)	WC (kA)	WT (ms)	HT (ms)	EF (kN)
500	7.5	560	600	4

Note: SQ is squeeze time, WC is welding current, WT is welding time, HT is hold time and EF is electrode force.

**Table 3:** Parameters and their levels for DPW scheme. (Reprinted from [26] with permission from Elsevier)

Parameters	Symbol	Unit	Levels			
			1	2	3	4
WC	A	kA	3	4.5	6	7.5
WT	B	ms	140	280	420	560
CT	C	ms	400	460	520	580

Note: WC is welding current, WT is welding time and CT is cooling time.

**Table 4:** Taguchi L16 (OA) for experimental design of DPW scheme.

Sample No.	A	B	C
1	1	1	1
2	1	2	2
3	1	3	3
4	1	4	4
5	2	1	2
6	2	2	1
7	2	3	4
8	2	4	3
9	3	1	3
10	3	2	4
11	3	3	1
12	3	4	2
13	4	1	4
14	4	2	3
15	4	3	2
16	4	4	1

**Table 5:** Experimental results of response characteristics, i.e., mean peak load and mean failure energy with corresponding S/N ratios.

Sample No.	Response 1: Mean Peak Load (kN)	S/N Ratio	Response 2: Mean Failure Energy(J)	S/N Ratio
1	24.63	27.83	68.35	36.70
2	24.64	27.83	74.23	37.41
3	24.70	27.85	79.01	37.95
4	25.58	28.16	81.71	38.25
5	24.67	27.84	77.57	37.79
6	25.11	28.00	81.20	38.19
7	26.00	28.30	87.51	38.84
8	26.55	28.48	91.76	39.25
9	24.81	27.89	81.14	38.19
10	25.50	28.13	87.90	38.88
11	26.74	28.54	98.59	39.88
12	27.00	28.63	100.38	40.03
13	25.83	28.24	88.92	38.98
14	26.76	28.55	99.52	39.96
15	27.06	28.65	102.53	40.22
16	27.14	28.67	105.46	40.46

**Table 6:** Parameter ranking for S/N ratios of peak load.

Level	A	B	C
1	27.92	27.95	28.26
2	28.16	28.13	28.24
3	28.30	28.34	28.19
4	28.53	28.48	28.21
Delta	0.61	0.53	0.07
Rank	1	2	3

**Table 7:** ANOVA for peak load.

Parameters/Factors	DF	Seq SS	Adj SS	Adj MS	F	P	Contribution (%)
A	3	0.78314	0.78314	0.261048	11.28	0.007 *	49.34
B	3	0.65401	0.65401	0.218003	9.42	0.011 *	41.2
C	3	0.01105	0.01105	0.003684	0.16	0.920	0.7
Residual Error	6	0.13886	0.13886	0.023144			8.7
Total	15	1.58707					100
R-Sq = 91.25%, R-Sq(adj) = 78.13%							

* Significant.

**Table 8:** Parameter ranking for S/N of failure energy.

Level	A	B	C
1	37.58	37.91	38.81
2	38.52	38.61	38.86
3	39.24	39.22	38.84
4	39.90	39.50	38.74
Delta	2.33	1.58	0.13
Rank	1	2	3

**Table 9:** ANOVA for failure energy.

Parameters/Factors	DF	Seq SS	Adj MS	F	P	Contribution(%)
A	3	11.9659	3.98864	105.25	0.000 *	65.82
B	3	5.9487	1.98290	52.32	0.000 *	32.72
C	3	0.0361	0.01202	0.32	0.813	0.19
Residual Error	6	0.2274	0.03790			1.25
Total	15	18.1781				100
R^2^ = 98.75%, R^2^(adj) = 96.87%						

* Significant.

## Data Availability

Data is contained within the article and available on reasonable request from the corresponding author.
